# Secondary Acute Myeloid Leukemia in Myelodysplastic Syndrome Patients Aged Over 60 Years

**DOI:** 10.7759/cureus.40124

**Published:** 2023-06-08

**Authors:** Dipabali Chaudhuri, Kokab Irfan Khan, Roba Al Shouli, Akhil Allakky, Asila A Ferguson, Aujala Irfan Khan, Baraa Abuzainah, Sai Dheeraj Gutlapalli, Pousette Hamid

**Affiliations:** 1 Research, California Institute of Behavioral Neurosciences & Psychology, Fairfield, USA; 2 Pediatrics, California Institute of Behavioral Neurosciences & Psychology, Fairfield, USA; 3 Internal Medicine, California Institute of Behavioral Neurosciences & Psychology, Fairfield, USA; 4 Psychiatry, California Institute of Behavioral Neurosciences & Psychology, Fairfield, USA; 5 General Practice, California Institute of Behavioral Neurosciences & Psychology, Fairfield, USA; 6 Neurology, California Institute of Behavioral Neurosciences & Psychology, Fairfield, USA

**Keywords:** bone marrow, allogenic stem cell transplant, hypomethylating agents, chemotherapy, leukemia, secondary acute myeloid leukemia, myelodysplastic syndrome

## Abstract

In myelodysplastic syndrome (MDS), neoplastic cells originate in hematopoietic stem cells of the bone marrow, causing dysplasia in multiple cell lines. This may ultimately lead to cytopenia and anemia. MDS generally occurs in patients aged over 60 years, and if left unchecked, it can lead to secondary acute myeloid leukemia (AML), which has a worse prognosis than de novo AML. Hence, it is important to find methods to treat and manage MDS and prevent secondary AML. This review tries to point out the best methods to find out the best possible treatment for MDS, which can lead to its remission or possibly cure and prevent it from progressing into AML. In order to do this, the pathogenesis of MDS is taken into account, and it is clear that the various molecular mutations that lead to the hematologic neoplasms directly affect the different chemotherapy agents that can be used. The different common mutations leading to MDS and secondary AML have been reviewed along with the drugs best inclined to target them. Some mutations lead to a worse prognosis than others, and ongoing mutations can lead to drug-resistant neoplasms. Thus, drugs targeting the mutations need to be used. The feasibility of an allogeneic stem cell transplant is also taken into account, as this can lead to a total cure of MDS. Methods of decreasing post-transplant recovery time and complications have been looked into, and more studies need to be done on the matter. Currently, it is clear that a more personalized approach to each individual case with its own set of drug combinations is the best approach to treating MDS and secondary leukemia and increasing the overall survival (OS).

## Introduction and background

Myelodysplastic syndromes (MDSs) are a group of disorders characterized by bone marrow neoplasm originating from hematopoietic stem cells, causing dysplasia in one or multiple cell lines, cytopenia, and ineffective hematopoiesis [1]. Acute myeloid leukemia (AML) is a type of myeloid neoplasm defined by an exaggerated amount of myeloblasts. There is a thin arbitrary line of 20% blast count separating AML from MDS. If a patient has less than 20% blasts, they are considered to have MDS, and if the count is 20% or more, it is considered to be AML with myelodysplastic changes (AML-MRC). This, however, is more of a guide than an absolute, and individual cases should be classified on the basis of a combination of their genetics, blast percentage, dysplasia and cytology [2]. For instance, there are often patients with only 15%-16% blast count who are categorized and treated under AML. Diagnosis and classification of MDS are mostly based on blast percentages, mutations, cytogenetics, and the degree of dysplasia, for example MDS with single lineage or multilineage dysplasia, MDS with ringed sideroblasts, MDS with excess blasts, MDS with isolated del(5q), or MDS that is unclassifiable [3]. MDS generally occurs in older adults (over 60-70 years old) and in people with a history of ionizing radiation, chemotherapy, or environmental factors such as benzene. On occasion, it can also be idiopathic [4].

MDS is coined as preleukemia by some as it has the possibility of transforming into leukemia, most commonly AML. This is what we call secondary AML or AML-MRC, and it tends to be much harder to treat than primary AML [4]. It is also important to note that a diagnosis of AML-MRC can be made based on the presence of MDS-related cytogenic abnormality even without a previous diagnosis of MDS [3]. MDS can occur due to mutations in genes such as ASXL1, BCL-6, BCOR, ETV6, STAG2, U2AF1, and ZRSR2 accumulating over time [5]. Overlapping mutations occur in AML, and hence, both disorders seem to share a similar natural course. However, the genetics of some MDS and AML patients show no cytogenic abnormalities, indicating that other molecular pathologies may also be causal to these disorders [6]. Secondary AML due to MDS tends to have the same genetic mutations as MDS. For example, FLT3-ITD mutation is more commonly seen in primary AML, whereas ASXL1, ETV6, and SRSF2 mutations are more commonly seen in MDS/secondary leukemia patients [5,6]. At the same time, though primary AML and MDS do share similar gene aberrations, there are also plenty of differences, such as MDS cells needing additional genetic mutations in order to develop into AML blasts (mutations in RUNX1, NRAS, and NTRK3) [7]. Since the pathophysiologies of secondary AML and MDS are so entwined, to have a better picture of them, it is necessary to know about the various genetic mutations and other molecular pathologies that lead to them. Only then can we begin to understand what causes the shift from MDS to secondary AML and how to cure or perhaps even prevent it.

In this review, we aim to discuss the various pathogenic processes leading to MDS and secondary AML. This will provide a better picture of what medications to implement in order to target the various causes of MDS and secondary AML. It will also help understand the reasons behind using various medications to treat MDS and secondary AML in different patients. It is also important to explore the effects of drugs used to treat hematologic neoplasms such as idarubicin, glasdegib, cytarabine, and APR-246 on MDS patients and secondary AML patients [8-10] and discuss the effects of these medications on younger patients versus much older patients. Along with chemotherapy, other treatment options such as various antiangiogenic agents and procedures such as radiation and bone marrow transplants are major avenues that can be used in MDS and secondary AML patients depending on the severity of the disease and the general health of the patient. For example, the feasibility of stem cell transplants in patients over 65 years is a major question. An allogeneic stem cell transplant, if available, can fully put a patient into remission; however, whether or not an older patient with comorbidities can physically and mentally handle the treatment is also something that has to be taken into account [11,12]. Finally, it is important to try and get consensus on the best possible means of treating MDS and secondary AML and perhaps even preventing secondary AML.

## Review

MDS occurs due to mutations accumulating over time. Some of the frequent mutations are *SETBP1, SF3B1 ASXL1, RUNX1, TP53, BCL-6, BCOR, ETV6, STAG2, U2AF1*, *ZRSR2,* and RAS/MAPK pathway mutations. They are normally associated with previous chemotherapy and radiation but can also occur due to idiopathic causes. As mentioned earlier, MDS subtypes are currently categorized on the basis of their genetics, cytogenetics, blast count, and dysplasia. Some of the common types are given in Table 1 [13].

**Table 1:** Common MDS subtypes with their corresponding mutations, blast counts, cytogenetics, and dysplasia. NOS: not otherwise specified; VAF: variant allele frequency; MDS: myelodysplastic syndrome; AML: acute myeloid leukemia.

	Dysplasia	Cytopenia	Blasts	Cytogenetics	Mutations
MDS-*SF3B1*	One or more than one	One or more than one	<5% bone marrow <2% peripheral blood	Any, except isolated del(5q), −7/del(7q), abn3q26.2, or complex	*SF3B1* (≥10% VAF), without multi-hit *TP53*, or *RUNX1*
MDS with del(5q)	One or more than one	One or more than one	<5% bone marrow <2% peripheral blood	del(5q), with up to 1 additional, except −7/del(7q)	Any, except multi-hit *TP53*
MDS, NOS without dysplasia	Zero	One or more than one	<5% bone marrow <2% peripheral blood	−7/del(7q) or complex	Any, except multi-hit *TP53* or *SF3B1* (≥10% VAF)
MDS, NOS with single lineage dysplasia	One	One or more than one	<5% bone marrow <2% peripheral blood	Any, except not meeting criteria for MDS-del(5q)	Any, except multi-hit *TP53; *not meeting criteria for MDS-*SF3B1*
MDS, NOS with multilineage dysplasia	Two or more than two	One or more than one	<5% bone marrow <2% peripheral blood	Any, except not meeting criteria for MDS-del(5q)	Any, except multi-hit *TP53,*; not meeting criteria for MDS-*SF3B1*
MDS with excess blasts-1 (MDS-EB-1)	One or more than one	One to three	5%-9% bone marrow 2%-9% peripheral blood, no Auer rods	Any	Any, except multi-hit *TP53*
MDS/AML	One or more than one	One to three	10%-19% bone marrow 5%-19% peripheral blood, or Auer rods	Any except AML defining	Any, except *NPM1*, bZIP *CEBPA,* or *TP53*

Other than the abovementioned type, there is another subtype known as MDS with mutated *TP53*. This subtype is categorized together along with MDS/AML with mutated *TP53* and AML with mutated *TP53*. These three are grouped together as they are all aggressive despite different blast percentages. These diseases have multi-hit *TP53* mutations, which correspond to a highly aggressive disease with short survival [13].

Even though MDS can often lead to secondary AML, that does not necessarily mean that all types of MDS will change into AML [5]. For instance, MDS with excessive blasts and MDS/AML have the biggest possibility of changing into AML. It is also evident that patients with specific mutations are more prone to deteriorating into AML than others. Studies on the molecular pathologies of MDS and secondary AML patients have also shown that the transformation to AML occurs alongside mutations in FLT3 and RAS genes. The patient's karyotype keeps evolving over the duration of MDS and progresses to AML [14]. Mutations such as the myeloid transcription factor, the chromatin-modifying gene, the cohesion complex gene, the spliceosome complex, and most importantly the TP53 mutations are seen in a higher proportion in AML secondary to MDS. In fact, it has been shown in studies that TP53 mutation is by itself an independent risk factor leading to a poor prognosis in AML secondary to MDS patients. In addition to the distinct statistics seen in terms of the decline in the overall survival (OS) rate of the patients with this mutation, it also increases the chances of MDS patients getting secondary AML [15]. TP53 mutation has also been positive in older patients (above 65 years old) more commonly in comparison with their younger counterparts, which adds to the statistics of poor prognosis and diminished relapse-free survival (RFS) rates in MDS-AML patients above 65 years of age [6]. There are also some protective genes that prevent the transformation of MDS into full-blown AML. *P300* is one such gene, as it potentiates cell differentiation. Loss of the gene causes downregulation of differentiation and signal transduction which can cause a sudden transformation from MDS to AML [16].

Diagnosing secondary AML uses a combination of blast count and cytogenetics. As mentioned earlier, a blast count of more than 20% still falls under AML, so if a previous MDS patient has an increase in blast count of >20% it will be considered secondary AML. But, at the same time if a patient who has been diagnosed with MDS with a blast count of between 10% and 19% (MDS/AML) shows AML defining cytogenetic abnormalities, he will also be categorized as having AML despite the lower blast count [3,13]. Table 2 shows the cytogenetic abnormalities that can be diagnostic of AML-MRC in >20% of blast cases.

 

**Table 2:** Cytogenic abnormalities in AML-MRC when >20% blasts. AML-MRC: acute myeloid leukemia with myelodysplastic changes. Source: Arber et al. [3].

Cytogenetic abnormalities
Complex karyotype (three or more abnormalities)
Unbalanced abnormalities
−7/del(7q)
del(5q)/t(5q)
i(17q)/t(17p)
−13/del(13q)
del(11q)
del(12p)/t(12p)
idic(X)(q13)
Balanced abnormalities
t(11;16)(q23.3;p13.3)
t(3;21)(q26.2;q22.1)
t(2;11)(p21;q23.3)
t(1;3)(p36.3;q21.2)
t(5;12)(q32;p13.2)
t(5;7)(q32;q11.2)
t(5;17)(q32;p13.2)
t(5;10)(q32;q21.2)
t(3;5)(q25.3;q35.1)

Some mutations can certainly lead to more severe disease and a greater chance of transition to secondary AML in MDS patients. But that does not mean that having multiple mutations will mean a worse prognosis [3,13]. Multilineage dysplasia with several mutations has been shown to have a much better outcome in comparison with MDS and AML having certain specific mutations such as *ASXL1* and *BCOR*. So, a case-by-case approach depending on the cytology and genetics of the particular patient, gathered by next-generation sequencing (NGS) or polymerase chain reaction (PCR), must be taken. This way, optimal therapeutic options can be chosen.

Other than the various mutations and cytogenic abnormalities, factors such as bone marrow microvessel density (MVD) and the presence of "cytotoxic-granzyme-B-positive T-cells" also determine the severity of the disease and which medications to be used. It is seen that in secondary AML, cases showing high bone marrow MVD generally have a poorer prognosis. This is probably because rampant angiogenesis supports faster stem cell proliferation and neoplasm growth [9,10].

Currently, various medications are being investigated and are being used to target specific mutations leading to MDS and secondary AML. Previously, MDS and AML were being treated with high-dose cytotoxic chemotherapeutic agents with severe adverse effects and stem cell transplant whenever possible. Using high-dose cytotoxic agents is, however, not recommended in older frail patients (over 60-70 years old) due to higher mortality rates at induction in older patients and patients with comorbidities. Hence, hypomethylating agents (HMAs) in combination with other drugs are being used, as the remission and cure rates of HMAs alone are still not optimal [17]. For instance, "Decitabine," a pyrimidine nucleoside analog, was considered a potential treatment for TP53 mutation-based MDS and secondary AML [6]. It is a DNA HMA that induces erythroid differentiation and increases levels of fetal hemoglobin [18]. Unfortunately, after several clinical trials, no conclusive statistical significance was seen when treating patients with secondary AML [7]. Regardless, the Decitabine trials helped determine the various markers of AML remission and relapse, such as the percentage of various hemoglobin in the body, which is still used to determine whether a patient will stay in remission or relapse [18]. Recently, an oral form of Decitabine in combination with cytidine deaminase (CDA) inhibitor Cedazuridine, termed C-DEC, has been approved as the first oral HMA, and its possible role in MDS and secondary AML along with other hematologic malignancies is of active interest [19].

Azacitidine, another HMA, has also been the mainstay of MDS-AML treatment in the elderly. Azacitidine, when compared to Decitabine, has shown better OS and is still being used as first-line treatment for MDS in older patients due to its relative safety and efficacy [19]. Unfortunately, failure of these agents in treating MDS often leads to disease progression to AML with a much worse prognosis [20]. This is probably due to the neoplastic cells gaining resistance against the drugs after multiple infusions. Hence, when the hypomethylating drugs fail, MDS patients should be put on individualized drug trials in order to tackle the problem early and to prevent disease progression to secondary AML resistant to most chemotherapeutic agents [21]. Now many other treatment options with less adverse effects and possibly better RFS are also being experimented with.

In order to figure out better treatment options for secondary AML, it is very important to understand that it is biologically different from de novo AML. Characteristics such as subclonal heterogeneity, upregulation of antiapoptotic proteins, and multidrug resistance are unique to secondary AML and thus make it much more of a challenge to treat [4]. In these situations, combination chemotherapy often has to be given. Previously "Cytarabine" (a pyrimidine analog that inhibits DNA polymerase) used to be given as chemotherapy for patients with both de novo AML and secondary AML, but the OS would be much lower in secondary AML patients. Now clinical trials have shown that using low doses of cytarabine (LDAC), rather than higher dosage as done previously, in combination with drugs like glasdegib (hedgehog pathway inhibitor) can increase OS and have shown a 22.8% decrease in death in secondary AML patients [4,8]. Other drug combinations, such as "CPX-351," a combination of "Daunorubicin and Cytarabine" [5,11], and "Glasdegib and Azacitidine," have also shown promise [22,23]. CPX-351 is in fact the only drug that has been specifically approved for AML-MRC. It has shown increased OS in comparison with standard HMAS therapy in most patients, including patients aged 60-75 years [7].

The monoclonal anti-CD47 antibody "Magrolimab" is currently being used to target MDS and AML neoplasms. It works by targeting "cluster of differentiation (CD) 47," enhances phagocytosis, and causes tumor growth inhibition [6,17]. Another novel drug "APR-246," which works as a TP53 reactivator, has been recently approved for use. It targets the mutant TP53 gene, is being used in MDS and certain AML patients, and continues to show promise. Apr-246 can restore the TP53 gene back to its wild variant and can help induce ferroptosis, a type of iron-dependent apoptosis. Some studies have also shown that APR-246 induces ferroptosis in neoplastic cells even without TP53 mutation [6]. Nevertheless, further studies need to be done to figure out which type of MDS/secondary AML patients will benefit more by APR-246. Moreover, there is a dearth of data on the effects of ferroptosis on AML, and APR-246 is the first ferroptosis-inducing agent being researched in oncology [6].

As mentioned earlier, increased bone marrow MVD worsens the prognosis Thus, antiangiogenic medications in combination with traditional chemotherapy agents are useful in treating cases with high MVD [9,10]. The drug "Lenalidomide" has shown prominent antiangiogenic effects in secondary AML and is used with success in "deletion of the long arm of chromosome 5 (del 5q)" MDS and AML. Del5q, the most common isolated mutation in MDS and AML, normally has an extremely poor prognosis. Lenalidomide has shown promise, and its immunomodulatory and antiangiogenic effects are useful in del5q MDS and AML. It also has indirect effects on the bone marrow microenvironment, which seems to have chemotherapeutic effects, but the exact method by which this occurs is unclear and needs further research. It has to be used with caution in the elderly, as excessive inhibition of angiogenesis can cause dangerous adverse effects such as internal bleeding.

**Figure 1:**
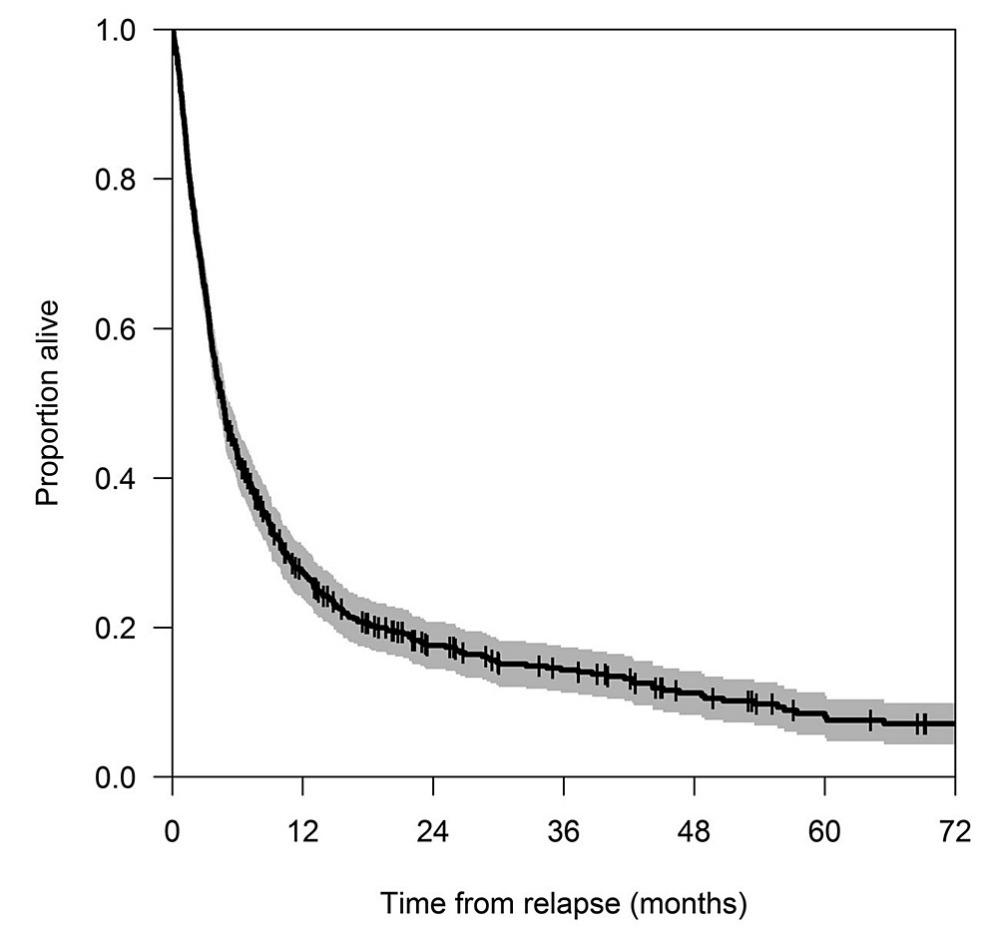
OS from relapse in a cohort of 698 patients. The gray area denotes 95% CI over time. OS: overall survival; CI: confidence interval. Source: Schmid et al. [26].

One of the definitive treatments that can put MDS and secondary AML patients into remission is an allogeneic stem cell transplant. It has been used more and more over the last few decades, and factors such as patient selection and post-transplant care have become much better over time. Unfortunately, for older patients (above 65 years), a stem cell transplant is generally not recommended as they are considered unfit and too fragile to withstand the procedure. However, recent studies show that, when selected appropriately, the average older patient's OS increases after transplant [24]. Even so, post-transplant relapse has been seen in MDS and secondary AML patients. One of the most significant risk factors for post-transplant relapse is the severity of the disease [25,26]. Preconditioning with chemotherapy and decreasing blast count before the transplant is one of the ways to increase OS and change the prognosis for the better. In MDS and secondary AML with CD34+/CD38- high cell burden, the prognosis and survival rates tend to be very low especially in older patients above 65 years even with stem cell transplant. But it is being seen that a combination of pre-transplant conditioning with drugs like busulfan + cyclophosphamide and busulfan + fludarabine has shown shorter recovery periods and decreased rates of relapse post-transplant [27]. Other than this, there are age-independent factors, such as leukocyte telomere length, that can determine post-transplant OS regardless of age. So, if the pre-transplant leukocyte telomere count is short, it can mean a poorer recovery post-transplant regardless of age, and pre-transplant longer leukocytic telomere length can mean a better post-transplant prognosis [28]. For relapse after the transplant, no standard approach has been established. Studies have been done on patients with post-transplant relapse to observe survival rates. In one such study involving a cohort of 698 patients, the effects of donor lymphocyte infusion and secondary stem cell transplant were observed. The median OS from relapse was recorded to be 4.7 months (95% confidence interval (CI): 4.1-5.3 months) [26]. Figure 1 demonstrates the OS from relapse observed in the previously mentioned group of 698 patients post stem cell transplant.

## Conclusions

It is clear from the clinical trials and studies reviewed above that understanding and studying the genetic changes in MDS and AML are extremely important. Multiple clinical trials have shown that knowing the specific mutation causing the neoplastic growth and then using a chemotherapeutic agent is much more effective and usually gives a better prognosis. Finally, it is important to understand that despite the risks, the best way to completely cure MDS and prevent secondary AML is a stem cell transplant when probable, along with pre- and post-transplant therapies to decrease post-transplant recovery time and complications, thus making bone marrow transplants in older patients more feasible. A thorough genetic investigation of MDS and secondary AML patients needs to be done to help categorize patients according to the various risk factors and prognoses. Currently, an increased number of research is being done on the individualization of treatment based on molecular mutations in patients in the hopes that this can improve patient response and OS and become the mainstay of future treatments.
